# Role of N6-methyladenosine methylation in glioma: recent insights and future directions

**DOI:** 10.1186/s11658-023-00514-0

**Published:** 2023-12-11

**Authors:** Chunlin Li, Bowen Li, Hui Wang, Linglong Qu, Hui Liu, Chao Weng, Jinming Han, Yuan Li

**Affiliations:** 1 Department of Neurology, Affiliated Hospital of Shandong University of Traditional Chinese Medicine, Jinan, 250014 Shandong China; 2 College of Traditional Chinese Medicine, Shandong University of Traditional Chinese Medicine, Jinan, 250000 Shandong China; 3 Department of Acupuncture, Zaozhuang Traditional Chinese Medicine Hospital, Zaozhuang, 277000 Shandong China; 4 First School of Clinical Medicine, Shandong University of Traditional Chinese Medicine, Jinan, 250000 Shandong China; 5 Department of Neurology, Renmin Hospital of Wuhan University, Wuhan, Hubei Province China; 6 Department of Neurology and Neurosurgery, Montreal Neurological Institute, McGill University, Montreal, QC Canada; 7 Department of Neurology, Xuanwu Hospital, Capital Medical University, Beijing, 100053 China; 8 Key Lab of Chemical Biology (MOE), School of Pharmaceutical Sciences, Cheeloo College of Medicine, Shandong University, Jinan, 250012 Shandong China; 9 Suzhou Research Institute of Shandong University, Suzhou 215123, China

**Keywords:** N6-methyladenosine methylation, RNA, Glioma, Neurogenesis

## Abstract

Glioma is the most pervasive intracranial tumor in the central nervous system (CNS), with glioblastoma (GBM) being the most malignant type having a highly heterogeneous cancer cell population. There is a significantly high mortality rate in GBM patients. Molecular biomarkers related to GBM malignancy may have prognostic values in predicting survival outcomes and therapeutic responses, especially in patients with high-grade gliomas. In particular, N6-methyladenine (m6A) mRNA modification is the most abundant form of post-transcriptional RNA modification in mammals and is involved in regulating mRNA translation and degradation. Cumulative findings indicate that m6A methylation plays a crucial part in neurogenesis and glioma pathogenesis. In this review, we summarize recent advances regarding the functional significance of m6A modification and its regulatory factors in glioma occurrence and progression. Significant advancement of m6A methylation-associated regulators as potential therapeutic targets is also discussed.

## Introduction

Glioma collectively refers to a group of neuroepithelial tumors, which are most commonly diagnosed as primary intracranial malignant neoplasm [1]. In accordance with the 2021 World Health Organization (WHO) classification of the Central Nervous System (CNS) tumors [2], gliomas are mainly classified into four categories, namely adult diffuse gliomas, childhood diffuse low-grade and high-grade gliomas and localized astrocytoma [3, 4]. Integrating the most up-to-date histopathological features and molecular phenotyping of gliomas, a new classification standard and grading system, has recently been proposed [2, 5]. According to the gene molecular diagnosis of isocitrate dehydrogenase (IDH) [6], gliomas can be classified into wild-type and mutant IDH. The glioma with the IDH mutation showed the cytosine-phosphate-guanine (CpG) island methylation phenotype (G-CIMP) [7]. Based on the degree of DNA methylation, non-coding clusters of IDH mutations were further subdivided into two different subgroups: low G-CIMP and high G-CIMP groups. It was found that G-CIMP DNA methylation (GMI CIMP^+^) showed great predictive value in GBM [8].

Clinical symptoms of glioma include intracranial hypertension, cognitive dysfunction, and seizures. Patients with glioma usually suffer from headaches, vomiting, and vision loss due to the occupying effects of tumors. Furthermore, glioma can cause increasing cerebral compression, leading to dyskinesia and other sensory disturbances. It is thus necessary to simultaneously consider age, disease status, and other critical clinic-pathological factors to carry out multidisciplinary integrated treatments, including surgical resection, radiotherapy, chemotherapy, systematic therapy, and supportive treatments [9, 10].

A variety of RNA modifications have been reported so far in relation to glioma pathogenesis [11, 12]. Apart from the 5'-cap and 3'polyA modifications, N6-methyladenine (m6A) mRNA modification mediates more than 80% of all types of RNA methylation. The m6A methylation involves methylation modification at the sixth nitrogen atom of adenine and is mainly regulated by the "Writers", "Erasers" and "Readers" enzymes. Studies have shown that the m6A modification improves the processing speed of the precursor mRNA, assists in mRNA nucleation, and regulates several RNA-associated cellular mechanisms during embryonic development [13, 14]. Hence, any aberrant changes in the functionality of m6A modification-related factors can result in multiple types of pathogenic conditions in the CNS, such as gliomas [15].

To date, the pathomechanistic role of m6A modification in glioma-related cellular pathways remains unclear. In this study, we critically reviewed the relationship between m6A methylation and glioma pathogenesis and then provided potential therapeutic approaches for glioma by targeting m6A at the molecular level.

## M6A-methylation regulation

### The regulatory factors of m6A methylation

**Fig. 1:**
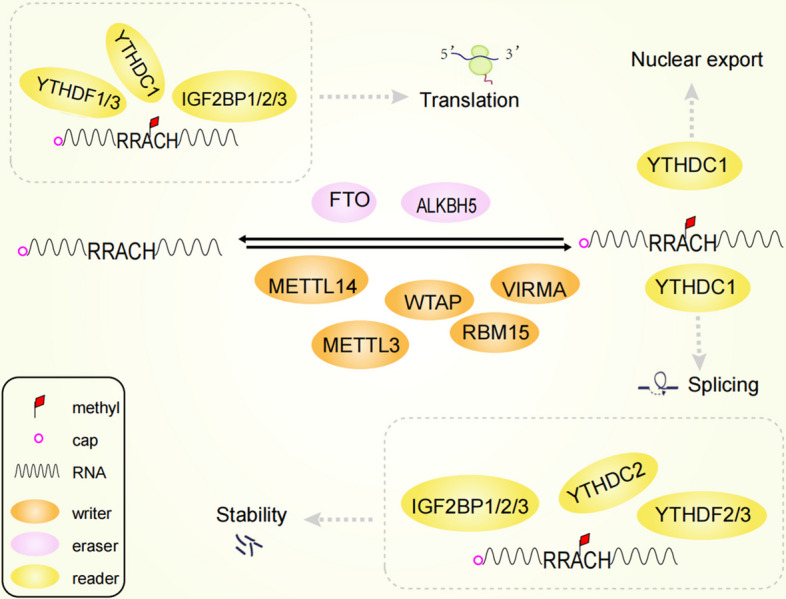
Dynamic and reversible processes of m6A methylation modifications. The m6A modification is primarily catalyzed by a methyltransferase complex, including METTL3, METTL14, WTAP, ZC3H13, etc. Demethylase FTO or ALKBH5 removes the m6A modification from the target mRNA. Reader proteins recognize m6A methylation and determine target mRNA fate

M6A methylation is the most characteristic post-transcriptional mRNA modification in eukaryotic cells [16], exerting its biological functions by modulating RNA metabolism [17]. The m6A modification occurs in the conserved RRACH (R, purine; H, non-guanine base; A, adenine; C, cytosine) motif [18], which is dynamically regulated by three classes of m6A methylases [19]. The m6A modification on the target mRNA is catalyzed by a methyltransferase complex, with the core catalytic proteins METTL3 and METTL14 and multiple additional regulatory proteins, including WTAP, RBM15/15B, VIRMA, and ZC3H13. METTL3 forms a stable core complex with METTL14 [20, 21] to catalyze the N6-methylation by transferring a methyl group from S-Adenosyl Methionine (SAM) to the adenosine of the target mRNA. While MELLT14 acts as an allosteric activator of METTL3,thereby, promoting efficient binding of the m6 methylation complex to the mRNA [22]. WT1-associated protein (WTAP) further improves this efficiency by recruiting the substrate and locating METTL3 and METTL14 in nuclear spots [23, 24]. The m6A methylation modification is reversible and can be reversed by RNA demethylases (Erasers), including Fat mass and obesity-associate protein (FTO) and Alk B homolog 5 (ALKBH5) [25], to maintain a dynamic balance between the methylated and non-methylated status of mRNA. In this process, the target recognition is accomplished by m6A binding proteins, called “Readers”, including the YTH domain family members, heterogeneous nuclear ribosomal protein (HNRNP) family proteins, and insulin-like growth factor 2 binding protein (IGF2BP). The m6A binding proteins modulate several RNA processing events such as stability [26], translation [27], splicing [28] and degradation [29] by directly or indirectly binding to the m6A modified mRNA. "Writers," "Erasers" and "Readers", as regulators of the m6A methylation, can affect mRNA transcription, translation, splicing, and stability of target genes at different levels [30, 31] (Fig. 1).

With the development of next-generation sequencing (NGS) and liquid chromatography (LC), the detection precision of m6A RNA modification has been improved significantly, even for challenging samples. Currently, available detection techniques for m6A methylation can be categorized into the following subtypes, based on the experimental goal, namely the transcriptome-wide identification and quantitation and high-throughput direct sequencing analysis of specific fragments or loci. The commonly used techniques for detecting the overall level of m6A modification include liquid chromatography (LC)–mass spectrometry (MS), dot blotting, and colorimetric assays. Molecular ion peaks and fragment ion peaks can be obtained using LC–MS carrying out qualitative and quantitative analysis of bases at the same time. The dot blotting and colorimetric assays are similar to LC–MS, while they are easier to operate. High-throughput sequencing techniques are methylated RNA immunoprecipitation-sequencing (MeRIP-Seq), m6A individual-nucleotide-resolution cross-linking and immunoprecipitation-sequencing (miCLIP-seq), and single-base resolution chips. The advantages of MeRIP-Seq include convenient, fast and low-cost features and can be used to analyze the hypermethylated mRNA regions qualitatively. However, MeRIP-Seq may only identify the region of m6A hypermethylation and cannot achieve the resolution of a single base. The method used for the first time to measure methylation levels of m6A at a high-throughput transcriptional level [32, 33] is called MeRIP-seq, which transforms mRNA fragments into 100nt-long oligonucleotides and enriches them with anti-m6A antibodies. Enriched mRNA fragments are then sequenced on a high-throughput sequencing platform. Furthermore, the miCLIP-seq [34] can identify methylation at a single base resolution, marking a big step forward in this field. Other techniques such as MeRIP-quantitative real-time-PCR (qRT-PCR), SELECT, and MazF-qPCR are also quantitatively used for single-base mapping of m6A methylation [35].

### M6A modification is involved in the regulation of the nervous system

In the mammalian brain, m6A methylation regulates neurogenesis. During neuronal development, expressions of METTL3, METTL14, and FTO proteins are increased along with the overall level of m6A methylation. It has been documented that METTL3 deletion significantly reduces m6A levels in adult neural stem cells (aNSCs), inhibiting their proliferation and resulting in developmental defects [36]. In *Mettl14*^−/−^ null mice, embryonic NSCs exhibit a significant decrease in their proliferative and differentiation capacities. It is suggested that METTL14-mediated m6A methylation dynamically regulates histone modification and affects gene expressions related to the proliferation and differentiation of NSCs [37]. In addition, conditional deletion of *Mettl14* resulted in smaller newborn pups and premature death before postpartum day 25 (P25). At the same time, these mice presented delayed cortical neurogenesis caused by reduced expression of PAX6 by radial glial cells. Similar phenotypes have also been observed in the brain of *Mettl3*^−/−^ knockout (KO) mice at the embryonic stage [38, 39]. Furthermore, *Mettl14* KO mice show a dramatic decrease in the number of neurons and delayed differentiation of different cortical neuron subtypes during cortical development [39]. Previous studies have revealed that FTO proteinopathy is associated with human obesity, playing an important role in regulating fat deposition and energy homeostasis [40]. FTO is highly abundant in the brain, where it regulates the proliferation and differentiation of embryonic NSCs through the PDGFRA/SOCS5-STAT3 axis. A recent study in the *Fto*^loxP/loxP^ mouse model has demonstrated that conditional ablation of Fto can lead to inhibition of adult neurogenesis and retarded neuronal development due to a decrease in neuron count in the brain [41]. Fto-deficient mice show decreases in their body weight and brain volumes, reduced expressions of anti-apoptotic genes and brain-derived neurotrophic factor (BDNF), and increased neuronal apoptosis [42]. These results confirm that m6A modification is necessary for normal brain development and function. Moreover, abnormal expressions of METTL3 and ALKBH5 can lead to RNA methylation imbalance, causing developmental defects (decreased numbers of Purkinje cells and increased apoptosis of cerebellar granule cells) in the cerebellum and ataxic phenotypes [43, 44]. In support of this phenomenon, decreased number of mature neurons and rapid cerebellar atrophy were found in *Alkbh5* KO mice [43].

The m6A methylation also regulates cognition, learning, and memory. For long-term memory, defects in the expression of immediate early genes (IEGs) can cause impaired learning ability and memory formation. It’s been found that METTL3 can enhance hippocampus-dependent memory consolidation by promoting the translation of early-response genes (ERGs) such as *Arc*, *Egr1,* and *c-Fos*. There might be a favorable relationship between the abundance of METTL3 and long-term memory consolidation, while an increased expression of METTL3 can significantly boost up learning efficiency [45]. In the striatum and striatum pallidus, the loss of *Mettl14* can result in serious adverse effects on learning response and reversal learning [46]. YTH N6-Methyladenosine RNA Binding Protein 1 (YTHDF1) contributes to the translation of m6A-methylated neuronal mRNAs in a neuronal-stimulus-dependent manner and increases the level of related proteins in long term potentiation (LTP) [47]. In contrast, FTO reduces hippocampal neurogenesis and thus suppresses hippocampus-dependent memory by inhibiting the translation of memory-promoting transcripts [48]. Deletion of the *Fto* gene enhances fear memory [48, 49], and causes neuronal dysfunction and behavioral abnormality via dopamine receptor type 2 (D2R) and type 3 (D3R)^50^. Besides, FTO deletion can lead to excessive activation of the hypothalamus–pituitary–adrenal (HPA) axis and inhibition of the BDNF signaling pathway in the hippocampus, thus resulting in the abnormal differentiation of hippocampal neurons, leading to the anxiety disorder and working memory impairment [51].

The m6A methylation modification also affects axonal growth in the CNS. The local translation of axonal mRNA plays a vital role in the growth and development of axons. FTO enriches in axons, where it catalyzes the demethylation of axonal RNAs (e.g., GAP-43 mRNA), regulates the local translation, and promotes axon elongation [52]. Importantly, m6A methylation modification also contributes to the neuronal repair and axonal regeneration following an injury. In the peripheral nervous system (PNS), axonal injury increases the level of m6A transcription and promotes mRNA translation and related protein synthesis through METTL14 and YTHDF1, thereby supporting axonal regeneration [53]. Furthermore, the renewal of injured retinal ganglion cells can also be achieved by m6A methylation in the CNS [53] (Table 1).

**Table 1:** The function of m6A methylation regulators in the central nervous system

Regulators	Roles in nervous system regulation	Related targets	References
METTL3	↑Proliferation of NSCs	↑Ezh2	[27]
	↑Cortical neurogenesis		[29]
	↑Cerebellar development	↓Atoh1, Cxcr4, Notch2	[35]
	↑Long-term memory formation	↑IEGs	[36]
METTL14	↑Proliferation of NSCs	↓CBP, p300	[28]
	↓Premature differentiation of NSCs	↓CBP, p300	[28]
	↑Cortical neurogenesis		[30]
	↑Acquisition of response learning and reversal learning	↑striatal mRNAs encoding neuron- and synapse-specific protein	[37]
	↑Axon regeneration	↑De novo gene	[44]
FTO	↑Proliferation and neuronal differentiation of aNSCs	↑PDGFRA/SOCS5-STAT3	[32]
	↓Neuronal apoptosis	↑BDNF	[33]
	↓Synaptic plasticity	↓ADRB2 / c-MYC / SIRT1	[39]
	↑Neuronal signaling	↑DA signaling pathway	[41]
	↑Hippocampal neurogenesis and cellular differentiation	↓HPA axis	[42]
	↑Axon elongation	↑GAP-43	[43]
ALKBH5	↑Cerebellar development	↓Mphosph9	[34]
YTHDF1	↑long-term synaptic plasticity	↑GRIN1, GRIN2A, GRIA1, CAMK2A, CAMK2B	[38]
	↑axon regeneration	↑De novo gene	[44]

↑Promotion, ↓Inhibition

### Role of m6A methylation in regulating carcinogenesis

**Fig. 2:**
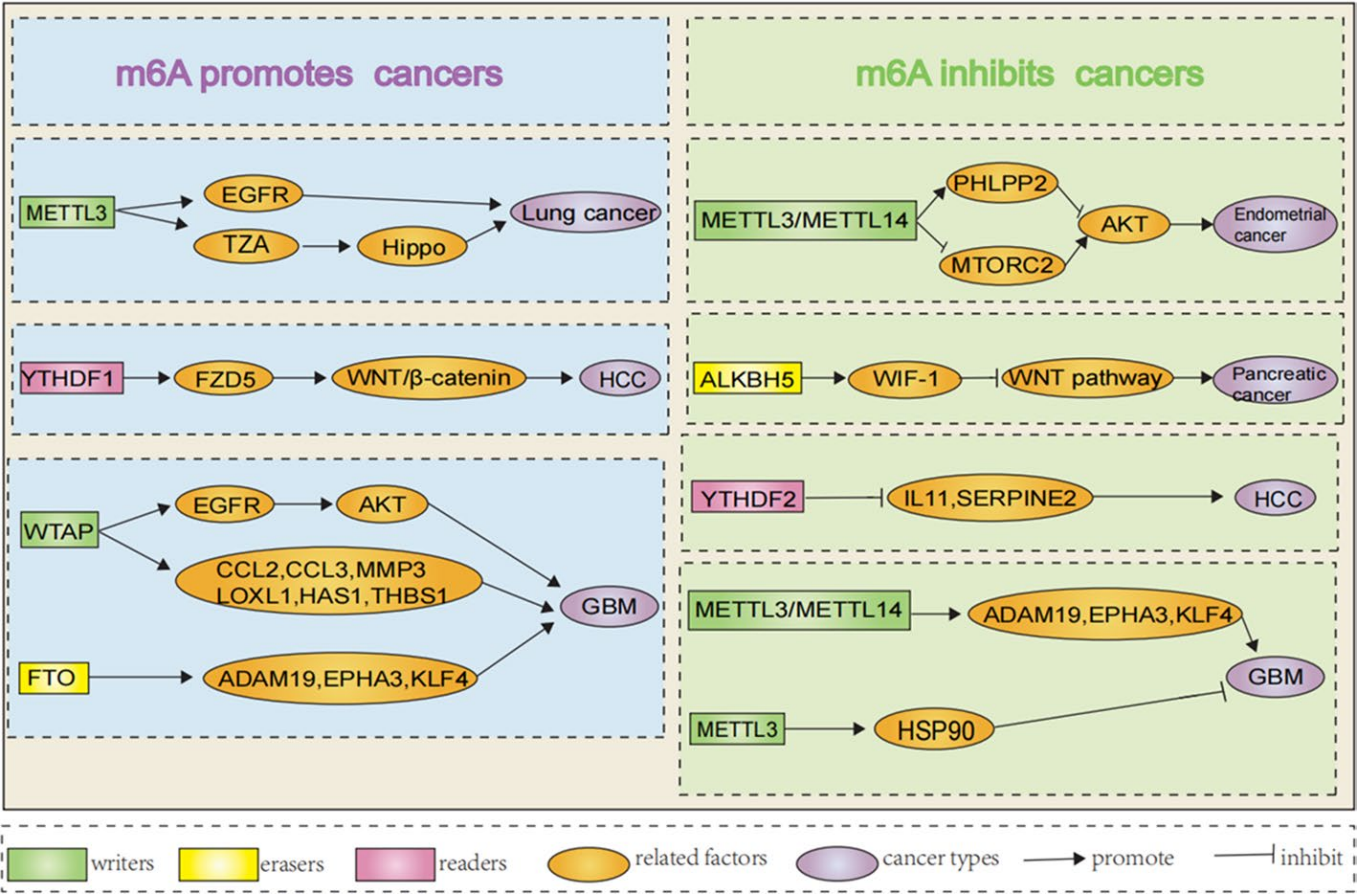
Example of functional m6A methylation-related regulators in cancers. M6A modification is a potential part of cancer progression by regulating the expression of tumor-related genes. M6A modification promotes cancer progression by enhancing the oncogene expression and inhibiting tumor-suppressor gene expression. M6A modification suppresses cancer progression by inhibiting oncogene expression while enhancing tumor-suppressor gene expression

The m6A modification plays a crucial role in regulating malignancy onset [54–56], and progression by altering the expression of tumor-related genes [57]."Writers" catalyze the m6A methylation of adenine on the target mRNA of the oncogene or tumor suppressor gene. "Readers" recognize the m6A methylation site and activate the downstream signaling pathways, resulting in a series of biochemical reactions enhancing the expression of oncogenes and reducing the expression of tumor suppressors. ‘Erasers’ remove the m6A methylation mark on the target mRNA and prevent the recognition or binding of "readers", thus increasing oncogene expression and/or decreasing tumor-suppressor genes’ expressions. The function of m6A modification is diverse in different tumors (summarized in Table 2 and Fig. 2). METTL3 plays a key role in the proliferation, survival, and invasion of lung cancer cells by promoting the expression of epidermal growth factor receptor (EGFR) and TAZ (a Hippo pathway effector) [58]. About 70% of endometrial neoplasms are associated with decreased m6A modifications. Decreased m6A modification is conducive to a decline in the expression of protein kinase B/Akt antagonist PHLPP2 and an increase in the expression of agonist mTORC2, thereby, promoting the growth and tumorigenicity of endometrial neoplasm cells [59]. Through regulating the Wingless/Integrated (WNT) pathway and reducing WIF-1 RNA methylation, ALKBH5 inhibits the tumorigenicity of pancreatic cancer cells. Deletion of *Alkbh5* has been shown to aggravate the occurrence and poor clinicopathological features of pancreatic cancers. Overexpression of ALKBH5 can reduce tumor proliferation, metastasis, and invasion activity in vitro and inhibit tumor growth in vivo [60]. Also, *Albkh5* KO promotes the progression of pancreatic cancer in the diseased animal model. YTHDF1 expression is significantly upregulated in hepatocellular carcinoma (HCC) and exhibits a positive correlation with pathological stages. YTHDF2 inhibits tumor cells and the tumor vascular system by regulating interleukin-11 (IL-11) and SERPINE2 mRNAs [61, 62].

**Table 2:** The function of m6A methylation regulators in common types of cancers

Disease	Regulator	Function	Role in disease	Related targets	References
Lung cancer	METTL3	Writer	Promote cancer	EGFR, TAZ	[49]
Endometrial cancer	METTL3/METTL14	Writer	Inhibit cancer	AKT pathway	[50]
Pancreatic cancer	ALKBH5	Eraser	Inhibit cancer	WIF-1	[51]
Hepatocellular cancer	YTHDF1	Reader	Promote cancer	FZD5, WNT/β-catenin pathway	[53]
	YTHDF2	Reader	Inhibit cancer	IL-11, SERPINE2	[52]
Glioma	METTL3	Writer	Inhibit cancer	HSP90	[55]
	METTL3/METTL14	Writer	Inhibit cancer	ADAM19, EPHA3, KLF4	[46]
	WTAP	Writer	Promote cancer	EGFR, AKT, CCL2, CCL3, MMP3, LOXL1, THBS1, HAS1	[57]
	FTO	Eraser	Promote cancer	ADAM19, EPHA3, KLF4	[46, 55]

Using large-scale glioma omics and clinical data from the Chinese Glioma Genome Atlas (CGGA) and the Cancer Genome Atlas (TCGA), researchers have found that primary regulators of the m6A methylation are differentially expressed in high-grade gliomas (WHO), and are closely related to the disease progression [63]. Li and colleagues have shown that the m6A methylation level is significantly decreased in glioma tissue, while its upregulation in U251 cells (which have mixed GSC properties [64]) leads to decreased cellular proliferation and migration [65] by modulating the HSP90 level. In support of this phenomenon, METTL3 or METTL14 downregulation has markedly promoted the proliferation, self-renewal, growth, and tumorigenesis of human glioblastoma stem cells (GSCs) [54]. On the contrary, overexpression of METTL3 restrains the proliferation and self-renewal of GSCs. WTAP is a nuclear protein associated with cellular proliferation and apoptosis regulation of GBM cells with being overexpressed in glioblastoma multiforme (GBM) [66, 67]. In summary, m6A methylation plays an essential biological role in the occurrence and development of gliomas and may serve as a potential therapeutic target for gliomas [12].

## Regulation of the m6A methylation "Writers" in glioma

### METTL3

GSC properties can promote the growth, invasion, and drug-resistant characteristics of GBM cells [68]. It has been found that METTL3 is overexpressed in GSCs, but gets downregulated during cellular differentiation [69, 70]. METTL3 promotes the maintenance of GSCs by controlling the mRNA stability of sex-determining region Y-box2 (SOX2). Furthermore, METTL3 promotes the development and self-renewal of GSCs by increasing the expression of stem-cell-specific marker (stage-specific embryonic antigen-1, SSEA1) and glioma reprogramming factors (POU3F2, OLIG2, SALL2, and SOX2) [12, 69]. However, other studies have provided an opposite conclusion regarding the role of METTL3 in GBM pathogenesis. *Mettl3* KD has been shown to enhance cellular proliferation, motility, and invasion of glioma cells in vitro, and glioma development in vivo [55, 65]. Potential reasons might include pathogenic mutations in other compensatory genes, epigenetic modifications, and high heterogeneity in the glioma cell population [71].

METTL3 plays an important regulatory role in RNA processing and oncogenic signaling. It has been recently recognized that METTL3 expression is correlated with the expression of Delta-Like Ligand 3 (DLL3), Notch Receptor 3 (NOTCH3), and Hairy and Enhancer of Split (HES1) in gliomas, indicating that METTL3 can directly activate the Notch signaling and increase the expression of downstream HES1 supporting glioma progression [72, 73]. Additionally, METTL3 regulates multiple carcinogenic pathways, such as the vascular endothelial growth factor (VEGF) and hedgehog signaling pathways. In contrast, the RAS, G protein-coupled receptor (GPCR), and cadherin signaling pathways are indirectly regulated by METTL3 [74]. IDH-wild type (WT) glioma is the most common type of astrocytoma. Chang and colleagues confirmed that the expression of METTL3 was positively correlated to an increased malignant grade of IDH-WT glioma [75]. METTL3 expression can enhance the stability of MALAT1 through the m6A methylation, subsequently activating the nuclear factor kappa-B (NF-κB) signaling, thus facilitating the malignant progression of IDH-WT glioma. METTL3 can also regulate the nonsense-mediated mRNA decay (NMD) pathway by increasing m6A methylation levels of serine- and arginine-rich splicing factors (SRSF) to promote glioma growth and invasion [76]. In addition, METTL3 KO promoted the susceptibility of GSCs to γ-irradiation [69]. These results suggest that METTL3 plays an important role in the pathogenesis of glioma, and METTL3-mediated m6A methylation is of great significance for GSC maintenance and radiotherapy resistance.

### METTL14

*METTL14* and *METTL3* are homologous genes. METTL14, a substrate recognition subunit, is a critical component of the m6A methyltransferase complex (MTC) [77]. METTL3 and METTL14 inhibit the GSC growth and self-renewal [14] by upregulating the expression of oncogenes such as ADAM metalloproteinase domain 19 (ADAM19), EPH receptor A3 (EPHA3), Kruppel-like factor 4 (KLF4) while downregulating the expression of tumor suppressors, including cyclin-dependent kinase inhibitor 2A (CDKN2A), breast cancer 2 (BRCA2) and tumor protein p53 inducible protein 11 (TP53I11).

ASS1, a tumor suppressor factor, participates in malignant incidences and disease progression by preventing the growth, migration, and invasion of glioma cells. METTL14 suppresses the ASS1 mRNA expression depending on the YTHDF2 expression. Therefore, the METTL14-ASS1-YTHDF2 signaling axis may serve as a promising therapeutic target for gliomas [78].

### WTAP

WTAP, identified as a splicing factor that binds to Wilms's Tumor-1 (WT1), is an active component of the MTC [79]. The expression of WTAP is significantly increased in gliomas, and is positively correlated with age, glioma grades [67], and adverse prognosis. Jin and colleagues have discovered that WTAP expression can be significantly upregulated in GBM, promoting the growth, migration, and invasion properties of GBM cells through the phosphorylation of EGFR and AKT [80]. WTAP may also function as a new therapeutic and prognostic marker [67]. GSCs promote GBM recurrence and therapeutic resistance by regulating intracellular microRNA (miRNA) biogenesis. For example, the overexpression of miR-29a could inhibit the WTAP expression in GSCs by decreasing the expression of QKI-6. In addition, miR-29a has been shown to prevent the proliferation, metastasis, and invasion of GSCs and promote cell apoptosis via regulating the 3-kinase (PI3K)/Akt signaling pathway [81]. We propose that WTAP may enhance the off-target activity of methyltransferases inducing GBM pathogenesis.

## Regulation of the m6A methylation "Erasers" in glioma

### ALKBH5

ALKBH5, belonging to the ALKB family, is a ferrous and 2-oxoglutarate-dependent nucleic acid oxygenase (NAOX). ALKBH5 plays a key role in regulating the proliferation [82], invasion [83], and migration [84] of tumor cells by catalyzing RNA demethylation. ALKBH5 is overexpressed in GSCs, enhancing cellular self-renewal, proliferation, and tumorigenicity. Forkhead box protein M1 (FOXM1), a member of the Forkhead transcription factor family, plays a critical role in modulating the cell cycle. Foxm1 regulates G1/S and G2/M transitions as well as the M phase progression [85], and its abnormal activation might associate with the growth and division of tumor cells. FOXM1 maintains the activity of GSCs by promoting β-catenin activation [86], interacting with MELK [87], inducing SOX2 expression [88], and activating STAT3 by phosphorylation [89]. FOXM1 mRNA 3'UTR is a unique methylation site for regulating the ALKBH5-FOXM1 interaction. Nuclear RNA binding protein HuR regulates the expression of FOXM1 nascent mRNA by binding unmethylated 3'UTR. In this case, 3'UTR methylation inhibits the interaction between HuR and FOXM1 nascent mRNA, resulting in decreased FOXM1 expression [56]. Furthermore, alternative splicing of FOXM1 mRNA can enhance the interaction between ALKBH5 and FOXM1 nascent RNA, inducing demethylation and then facilitating HuR binding and FOXM1 expression [56]. A recent study suggests that ALKBH5 is upregulated in glioma. Erasing the m6A methylation of glucose-6-phosphate dehydrogenase (G6PD) enhances its stability and then ALKBH5 promotes G6PD translation and activates the pentose phosphate pathway (PPP) to induce the proliferation of glioma cells [90].

ALKBH5 may serve as an important indicator to predict the prognosis of GBM patients, with a low expression of ALKBH5 being closely associated with prolonged overall survival. MiR-193a-3p, a tumor suppressor, targets ALKBH5 exerting an anti-tumor effect by restraining the growth of glioma cells and promoting apoptosis via inhibiting the AKT2 pathway [91]. Furthermore, the overexpression of ALKBH5 regulates the homologous recombination (HR) pathway to boost radio-resistance [92] and favors the invasion of GSCs to enhance GBM aggression [92, 93]. Therefore, ALKBH5 could be a potential therapeutic target for the radioresistance and aggressiveness of gliomas.

### FTO

FTO, first discovered as an m6A demethylase, is widely expressed in human tissue and cell types, as well as in other mammals. The expression of FTO is relatively high in the human brain, and it can contribute to tumorigenesis [94].

Approximately 80% of GBM and II-III grade gliomas bear somatic mutations in the *IDH1* gene [95]. The R132H mutation in IDH1 (IDH1-R132H) has the gain-of-function (GOF) effect causing the conversion of alpha-ketoglutaric acid (α-KG) to the oncometabolite D-2-hydroxyglutaric acid (D-2HG) during the oxidation of NADPH to NADP^+^ [96]. Previous studies suggest that excessive accumulation of D-2HG contributes to tumor initiation and progression [97, 98]. However, D-2HG may exert an anti-tumor effect by increasing the level of m6A methylation modification [99]. D-2HG suppresses the expression of FTO while increasing the level of m6A modification and inhibiting c-MYC expression [100]. It is demonstrated that D-2HG regulates the FTO/m6A/c-MYC/CEBPA signaling axis to exert antineoplastic effects by suppressing the growth and proliferation of tumor cells through FTO overexpression. C-MYC is a primary regulator of cell proliferation and is overexpressed in gliomas [101]. Xiao and colleagues have recently elucidated a c-MYC-miRNA-MXI1 feedback loop and found that c-MYC inhibits the expression of MXI1 via miR-155 and miR-23a clusters [102].

FTO overexpression enhances the stability of c-MYC mRNA through the m6A methylation-dependent mechanism, thereby improving MYC transcription, promoting the MYC feedback loop, and enhancing malignant characteristics of glioma cells [102]. Combined treatment with the FTO inhibitor MA2 [103] can improve the inhibitory effect of chemotherapeutic temozolomide (TMZ) on the proliferation and invasion of glioma cells [102]. A low expression level of FTO can also be associated with a worse prognosis. Mechanistically, FTO regulates the maturation of primary miR-10a via the m6A-dependent pathway to modulate glioma progression [104].

## Regulation of the m6A methylation "Readers" in glioma

### YTH domain family

The YTH domain family members are the first identified m6A methylation readers, including YTHDF and YTHDC [61]. YTH proteins have a conserved m6A binding domain that recognizes m6A modification through a conserved aromatic cage and two accessory proteins (FMR1 and LRPPRC). Among five proteins carrying the YTH domain, YTHDC1 is the only nuclear protein involved in transcription regulation, mRNA splicing, and nuclear export. YTHDF1, YTHDF2, YTHDF3, and YTHDC2 are the “reader” of m6A methylation and are engaged in mRNA translation and degradation processes [105].

The expression of YTHDF2 in glioma is highly associated with malignancy and disease prognosis. YTHDF2 specifically stabilizes the c-MYC mRNA in GSCs and facilitates the expression of downstream target IGFBP3 in an m6A methylation-dependent manner promoting the GSC growth [106]. EGFR is found constitutively active in most GBM cancers and phosphorylates YTHDF2 at serine39 and threonine381 via the EGFR-SRC-ERK signaling pathway to stabilize YTHDF2 protein. In GBM cells, YTHDF2 overexpression perturbs the cholesterol balance by downregulating LXRα and HIVEP2, and promoting the growth, invasion, metastasis, and tumorigenesis of GBM cells [107]. Chai and colleagues have found that YTHDF2 accelerates the degradation of UBX domain protein 1 (UBXN1) mRNA, inhibiting UBXN1 expression via the METTL3-mediated m6A methylation, that in turn activates the NF-κB signaling [108]. Activated NF-κB then stimulates STAT3, CEBPB, and TAZ by phosphorylation, enhancing the invasion, tumorigenesis, and therapeutic resistance of gliomas [109, 110]. YTHDF2 expression is significantly decreased in GBM, where it plays an indispensable role in the ASS1 mRNA decay process [78], thus facilitating the occurrence and development of GBM.

The carcinogenic effect of YTHDF1 has been observed in GBM. YTHDF1 downregulation results in decreased tumor sphere formation and compromised cancer stemness properties [111]. YTHDF1 deletion also causes decreased proliferation of GBM cells, while increasing the TMZ sensitivity [111, 112]. This study demonstrates that YTHDF1 controls malignant biological behaviors of GBM, including high proliferation rate, invasive nature, and chemo-resistance. In addition, it has been found that YTHDF1 is positively regulated by Musashi-1 (MSI1), a post-transcriptional regulator of gene expression correlated with a high degree of malignancy in GBM [111]. YTHDC1, involved in glioma occurrence, promotes GBM phenotypes depending on the m6A methylation site binding activity [76]. Furthermore, a YTHDC1-dependent mechanism has been proposed for the elimination of m6A methylation around the start codon of splicing factors [76]. These findings suggest that the YTH domain family proteins can play crucial roles in initiating glioma onset and progression through multiple pathways. Furthremore, eIF3, consisting 13 subunits, has the largest molecular weight and most complex structure of all eukaryotic initiation factors. eIF3 is related to translation initiation, termination, ribosomal cycle and stimulus-stop codon reading [113]. In the cytoplasm, YTHDF1 interacts with eIF3, binds to the m6A site around the stop codon, increases the transmission of the mRNA transcriptional complex and combines the translation initiation mechanism to promote translation initiation and protein synthesis [114, 115]. Liu and colleagues [116] found that most of the YTHDF1 binding sites (including eIF3C) were consistent with the m6A site by eCLIP-seq analysis. eIF3C is one of subunits where eIF3 coordinates the initiation factor and ribosome interaction for translation. Silencing YTHDF1 significantly decreased the expression of eIF3A and 3B in Merkel cell carcinoma cells [117], which further supported in the condition of melanoma [113, 118]. These results suggest that YTHDF1 combines with eIF3 at the beginning of translatability to induce translation initiation, promotes protein synthesis, and contributes to tumor occurrence and metastasis. However, there is limited research on the role of YTHDF1 and eIF3 in glioma.

### IGF2BP family

As post-transcriptional regulators, IGF2BP family proteins have been linked to glioma proliferation, invasion, and chemotherapy resistance. Both IGF2BP2 and IGF2BP3 are overexpressed in high-grade gliomas and indicate poor prognosis. IGF2BP2 binds to let-7 miRNA recognition elements (MREs) and suppresses the silencing effect of let-7 on its target genes, thereby maintaining the stemness of GSCs and promoting glioma development [119]. IGF2BP2 promotes GSC clonogenicity by regulating oxidative phosphorylation (OXPHOS) and maintaining glioma cells’ oxygen consumption rates [120]. Mu and colleagues have revealed that IMP2 controls IGF2 activity to stimulate the PI3K-AKT signaling pathway, thus facilitating the growth, metastasis, invasion, and epithelial-to-mesenchymal transition (EMT) of GBM cells [121]. Similarly, IGF2BP3 is not expressed in low-grade astrocytomas, while upregulated expression has been documented in GBM. IMP3 binds to the 5'-UTR of IGF2 mRNA, promoting IGF2 protein expression and activating the downstream PI3K-MAPK pathway. These pathways play an important role in promoting the growth, metastasis, invasion, and chemotherapy resistance of glioma cells [122]. Inhibition of IMP2 increases the sensitivity of GBM cells to TMZ therapy [121]. IGF2BP2 expression also associates with etoposide resistance in glioma cells [9]. Mechanistically, IGF2BP2 overexpression decreases DANCR methylation and increases its stability to inhibit FOXO1 ubiquitin-mediated PID1 expression, induce chemoresistance, and metastasis of GBM cells [123, 124].

IMP3 has been shown to overexpress in glioma cells, and its expression modulation has been linked to glioma grading. IMP3 overexpression also suppresses E-cadherin expression, while levels of N-cadherin, Vimentin, Snail, Slug, and MMP9 increase. IMP3 induces EMT in GBM to promote cellular proliferation, migration, and invasion [125]. p65, a subunit of the heterodimer of nuclear factor-κ B, is an essential regulator of glioma cell migration promoted by IMP3. IMP3 can also enhance p65 expression and activate the NF-κB signaling pathway by specifically binding to sites on the p65 mRNA 3'UTR, which in turn promotes metastatic migration of glioma cells [126]. Further, IMP3 is involved in circular RNA (circRNA)-mediated carcinogenicity. In this context, circNEIL3 and circHIPK3 have been found to play important roles [127, 128].

Long non-coding RNAs (lncRNAs) can serve as promising targets for treating gliomas, with IGF2BP1 playing an important role in the inhibition of glioma. By modulating the miR-526b-3p/IGF2BP1/MAPK signaling, LINC00689 silencing inhibits glioma tumorigenesis in vivo [129]. Lnc-THOR silencing induces MAGEA6 degradation and AMPK activation to inhibit human glioma cell survival [130]. Furthermore, miR-4500 downregulates the level of IGF2BP1 and its downstream factors (Gli1, IGF2 and c-Myc), thereby suppressing the proliferation, migration, and invasion of human glioma cells through targeting IGF2BP1 mRNA 3'-UTR [131].

### hnRNPA2B1

**Fig. 3:**
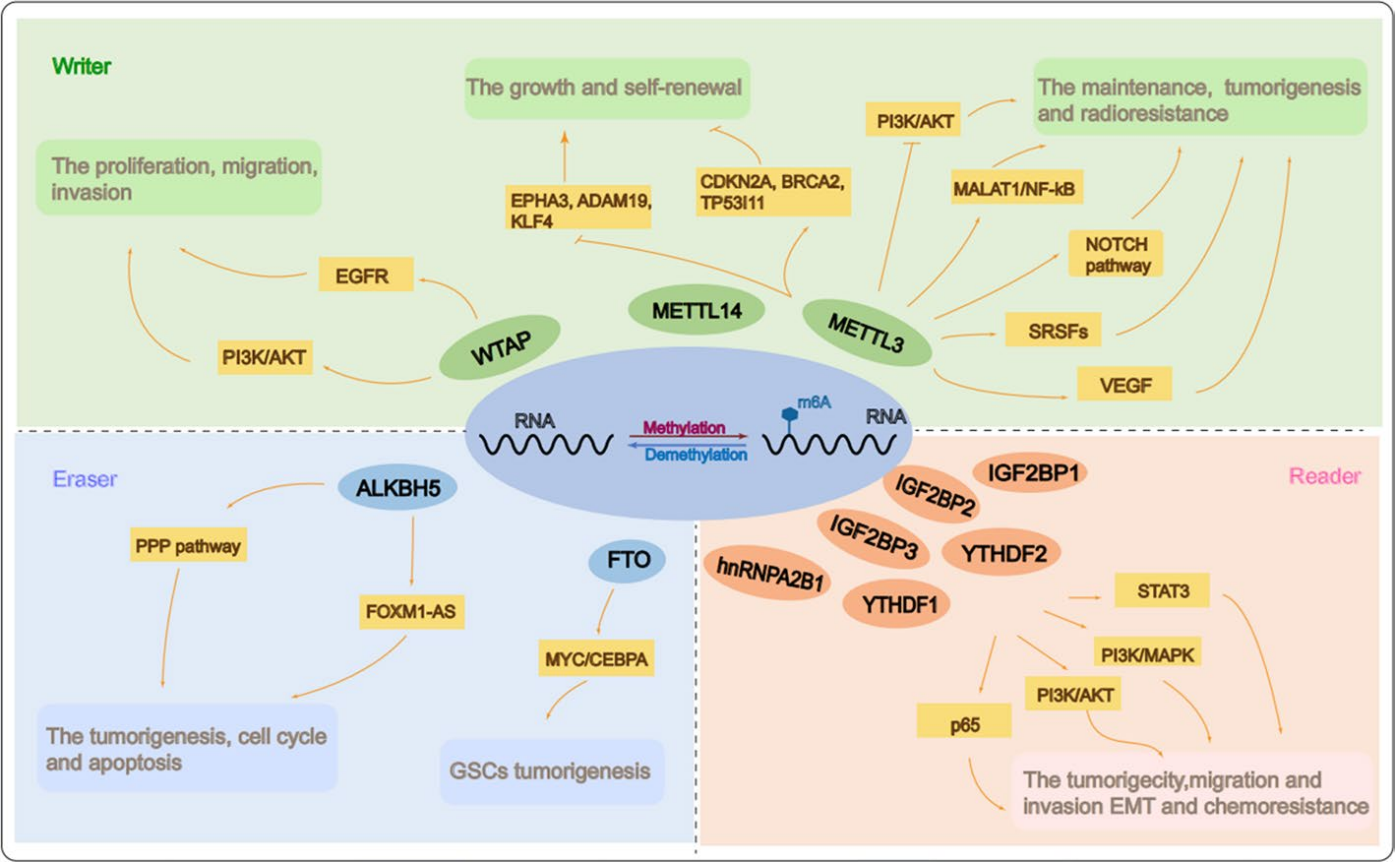
M6A methylation-related regulators in glioma. Regulatory factors of m6A methylation affect the growth, self-renewal, proliferation, differentiation, autophagy, apoptosis, migration, invasion, drug resistance, and immunosuppression of glioma stem cells (GSCs) through various cellular pathways to regulate the occurrence and development of gliomas

HnRNPA2B1 belongs to the hnRNP-binding protein family and serves as a reading protein in the m6A methylation modification process. Its downstream proteins are highly expressed in gliomas and are associated with glioma grading and poor survival [132, 133]. Regarding the role of IGF2BP3 in the carcinogenicity of circNEIL3, it has been found that circNEIL3 is packaged as an exosome by hnRNPA2B1 and transferred to infiltrate tumor-associated macrophages (TAMs), thus gaining immunosuppressive properties that promote glioma progression [128]. In support of this observation, hnRNPA2B1 KO has been shown to inhibit GBM proliferation, migration, and invasion. In addition, it can cause therapeutic resistance of TMZ, while inducing the production of reactive oxygen species (ROS) and apoptosis in the glioma U251 cell line [132]. Yin and colleagues have explored hnRNPA2/B1-mediated mechanisms in promoting glioma growth and they have found that hnRNPA2/B1 activates the AKT-STAT3 signaling pathway to increase expressions of B-cell lymphoma-2 (BCL-2), CyclinD1 and proliferating cell nuclear antigen (PCNA) [134]. β-Asarone, the major components of Shi Chang Pu, has been demonstrated to induce apoptosis and cell cycle stagnation in U251 cells by inhibiting the hnRNPA2/B1-mediated signaling pathways [135] (Tables 3 and 4, Fig. 3).

**Table 3:** M6A methylation-related regulators in GBM

Function	Regulators	Role in GBM	Related targets	References
Writer	METTL3	Promote	↑SOX2, SALL2, SSEA1, POU3F2, OLIG2	[60]
	METTL3	Inhibit	↓EPHA3, ADAM19, KLF4 ↑CDKN2A, BRCA2, TP53I11	[6]
	METTL3	Inhibit	↓PI3K/Akt pathway	[61]
	METTL3	Promote	↑NOTCH3, DLL3, HES1	[63]
	METTL3	Promote	↑VEGF	[65]
	METTL3	Promote	↑MALAT1, NF-κB	[66]
	METTL3	Promote	↑SRSF	[66]
	METTL14	Inhibit	↓EPHA3, ADAM19, KLF4 ↑CDKN2A, BRCA2, TP53I11	[6]
	METTL14	Promote	↓ASS1	[69]
	WTAP	Promote	↑EGFR	[71]
	WTAP	Promote	↑AKT, CCL2, CCL3, MMP3, LOXL1, THBS1, HAS1	[57]
Eraser	ALKBH5	Promote	↑Foxm1	[47]
	ALKBH5	Promote	↑G6PD, PPP	[82]
	ALKBH5	Promote	↑Rad51, XRCC2, BRCA2, EXO1, BRIP1	[84]
	FTO	Promote	↑MYC	[94]
Reader	YTHDF1	Promote	NA	
	YTHDF2	Promote	↑MYC, IGFBP3	[98]
	YTHDF2	Promote	↓LXRα, HIVEP2	[99]
	YTHDF2	Promote	↓UBXN1	[100]
	YTHDF2	Promote	↓ASS1	[69]
	IGF2BP1	Promote	↑MAPK	[115]
	IGF2BP2	Promote	↑let-7miRNA	[105]
	IGF2BP2	Promote	↑PI3K/Akt	[107]
	IGF2BP2	Promote	↑DANCR	[110]
	IGF2BP3	Promote	↑PI3K/MAPK	[108]
	IGF2BP3	Promote	↑p65	[112]
	IGF2BP3	Promote	↓E-cadherin	[111]
	IGF2BP3	Promote	↑N-cadherin, vimentin, snail, slug and MMP9	[111]
	hnRNPA2B1	Promote	↑AKT, STAT3 pathway	[120]

*NA* not applicable

**Table 4:** Molecules regulating the m6A methylation process

molecules	Regulatory roles	Target	References
QKI-6	Inhibitor	WTAP	[72]
R-2HG	Inhibitor	FTO	[92]
MA/MA2	Inhibitor	FTO	
Musashi-1	Regulator	YTHDF1	[103]
EGFR	Regulator	YTHDF2	[99]
miR-526b-3p	Inhibitor	IGF2BP1	[115]
miR-4500	Inhibitor	IGF2BP1	[117]

## Clinical significance of the m6A modification in glioma

The m6A methylation connects epitranscriptomics with tumorigenesis, playing a crucial role in affecting the growth, self-renewal, proliferation, differentiation, autophagy, apoptosis, migration, invasion, drug resistance, and immunosuppression of GSCs. Therefore, it is anticipated that related regulatory factors involved in the m6A methylation may serve as promising therapeutic targets.

Meclofenamic acid (MA) can specifically compete with FTO by binding to m6A modification sites. MA can significantly delay glioma development and increase the survival time of transplanted tumor-bearing mice [103]. MA, as an FTO inhibitor with high selectivity, offers a novel approach to glioma drug research. ALKBH5 and FTO belong to the 2OGX family and researchers have developed new 2OGX inhibitors based on the interacting domains of FTO and ALKBH5 [136, 137], that closely bind to the catalytic active sites. Developing more selective inhibitors of FTO and other m6A regulatory factors may help develop effective treatments for life-threatening gliomas. Combining these inhibitors and other therapeutic agents may overcome potential drug resistance for gliomas. Indeed, D-2HG exhibits a synergistic impact in combination with common therapeutic substances, including ATRA, AZA, Decitabine, and Daunorubicin [14]. The overexpression of ZDHHC17 in GBM contributes to disease development and malignant progression via the ZDHHC17-MAP2K4-JNK-p38 pathway [138]. Moreover, Genistein has been shown to exert a prospective curative effect on treating ZDHHC17-expressing GBM [138].

The expression of YTHDF1 is positively related to a poor prognosis in glioma patients [139]. WTAP is highly expressed in gliomas, especially in high-grade gliomas [67]. Expressions of WTAP, AL-BKH5, and YTHDF2 have been closely associated with the WHO grades. Nevertheless, FTO expression levels are positively related to the increasing malignancy of gliomas [63]. The risk scores derived from m6A regulatory factors can independently predict the survival of glioma patients. In addition, the Cox regression algorithm has been used to analyze 11 related prognostic genes, including *ALKBH5*, *YTHDF1*, *YTHDF2,* and *HNRNPC* in the CGGA to predict risk scores in glioma patients. In a study, according to the average risk rating, patients with glioma in CGGA and TCGA databases were split into the low-risk and high-risk groups, which showed a significant difference in overall survival (OS) between the two groups, and GBM patients with a low-risk rating were less sensitive to the TMZ treatment [63].

## Conclusions and perspectives

The m6A methylation modification regulates physiological processes such as neurogenesis, brain development, learning, and memory, thus playing an important role in the pathogenesis of glioma. Our understanding of the function of m6A modification in gliomas is still limited. First, it needs to be further clarified regarding the expression, function, and regulatory mechanisms of other m6A methylation regulators (including KIAA1429, RBM15) in gliomas. Second, the potential roles of m6A methylation regulators and molecular mechanisms are controversial, such as the "duality" of METTL3. Third, understanding the regulatory factors of m6A methylation may help us explore the pathological function of m6A modification in glioma.

Effective agents and new therapeutic strategies related to m6A modification are still in their infancy. We need to clarify disease-associated target mRNA and detailed regulatory mechanisms to provide new insights for glioma therapy.

Approaches to identify novel m6A modifications and differential expressions of m6A regulators in gliomas may benefit the early clinical diagnosis and prognostic predictions in glioma patients. FDA-approved FTO inhibitors can inhibit GSC growth and self-renewal. MA and D-2HG, as molecular factors aimed to regulate m6A factors, can be improved and applied to treat gliomas in an m6A-specific manner.

In summary, the level of m6A methylation and its regulatory factors are closely associated with the occurrence and disease progression in gliomas. The m6A methylation is expected to become a potential therapeutic target for gliomas in the near future.

## Data Availability

Not applicable.

## Abbreviations

CNS: Central nervous system
GBM: Glioblastoma
m6A: N6-methyladenine
WHO: World Health Organization
SAM: S-adenosyl methionine
WTAP: WT1-associated protein
HNRNP: Heterogeneous nuclear ribosomal protein
IGF2BP: Insulin-like growth factor 2 binding protein
NGS: Next-generation sequencing
LC: Liquid chromatography
HPLC: High-performance liquid chromatography
MS: Mass spectrometry
MeRIP-Seq: Methylated RNA immunoprecipitation-sequencing
miCLIP-seq: M6A individual-nucleotide-resolution cross-linking and immunoprecipitation-sequencing
qRT-PCR: Real-time-PCR
aNSCs: Adult neural stem cells
P25: Postpartum day 25
KO: Knockout
BDNF: Brain-derived neurotrophic factor
IEGs: Immediate early genes
ERGs: Early-response genes
YTHDF1: YTH N6-methyladenosine RNA binding protein 1
LTP: Long term potentiation
D2R: Dopamine receptor type 2
D3R: Dopamine receptor type 3
HPA: Hypothalamus–pituitary–adrenal
PNS: Peripheral nervous system
EGFR: Epidermal growth factor receptor
HCC: Hepatocellular carcinoma
IL-11: Interleukin-11
CGGA: The Chinese Glioma Genome Atlas
TCGA: The Cancer Genome Atlas
GSCs: Glioblastoma stem cells
DLL3: Delta-like ligand 3
NOTCH3: Notch receptor 3
HES1: Hairy and enhancer of split
VEGF: Vascular endothelial growth factor
GPCR: G protein-coupled receptor
WT: Wild type
NF-κB: Nuclear factor kappa-B
NMD: Nonsense-mediated mRNA decay
MTC: Methyltransferase complex
EPHA3: EPH receptor A3
KLF4: Kruppel-like factor 4
CDKN2A: Cyclin-dependent kinase inhibitor 2A
TP53I11: Tumor protein p53 inducible protein 11
WT1: Wilms's Tumor-1
miRNA: MicroRNA
NAOX: Nucleic acid oxygenase
FOXM1: Forkhead box protein M1
G6PD: Glucose-6-phosphate dehydrogenase
PPP: Pentose phosphate pathway
HR: Homologous recombination
GOF: Gain-of-function
α-KG: Alpha-ketoglutaric acid
D-2HG: D-2-hydroxyglutaric acid
TMZ: Temozolomide
UBXN1: UBX domain protein 1
MREs: MiRNA recognition elements
OXPHOS: Oxidative phosphorylation
EMT: Epithelial-to-mesenchymal transition
lncRNAs: Long non-coding RNAs
TAMs: Tumor-associated macrophages
ROS: Reactive oxygen species
BCL-2: B-cell lymphoma-2
PCNA: Proliferating cell nuclear antigen
MA: Meclofenamic acid
OS: Overall survival
